# The clinical and neurocognitive functional changes with awake brain mapping for gliomas invading eloquent areas: Institutional experience and the utility of The Montreal Cognitive Assessment

**DOI:** 10.3389/fonc.2023.1086118

**Published:** 2023-02-22

**Authors:** Yuan Wang, Shaochun Guo, Na Wang, Jinghui Liu, Fan Chen, Yulong Zhai, Yue Wang, Yang Jiao, Wenjian Zhao, Chao Fan, Yanrong Xue, GuoDong Gao, Peigang Ji, Liang Wang

**Affiliations:** ^1^ Department of Neurosurgery, Tangdu Hospital, Fourth Military Medical University, Xi’an, Shaanxi, China; ^2^ Department of Health Statistics, Fourth Military Medical University, Xi’an, Shaanxi, China; ^3^ National Time Service Center, Chinese Academy of Sciences, Xi’an, Shaanxi, China; ^4^ School of Optoelectronics, University of Chinese Academy of Sciences, Beijing, China

**Keywords:** glioma, awake brain mapping, extent of resection (EOR), Karnofsky Performance Status (KPS), neurocognitive status, Montreal Cognitive Assessment (MoCA)

## Abstract

**Objective:**

Awake craniotomy with intraoperative brain functional mapping effectively reduces the potential risk of neurological deficits in patients with glioma invading the eloquent areas. However, glioma patients frequently present with impaired neurocognitive function. The present study aimed to investigate the neurocognitive and functional outcomes of glioma patients after awake brain mapping and assess the experience of a tertiary neurosurgical center in China over eight years.

**Methods:**

This retrospective study included 80 patients who underwent awake brain mapping for gliomas invading the eloquent cortex between January 2013 and December 2021. Clinical and surgical factors, such as the extent of resection (EOR), perioperative Karnofsky Performance Score (KPS), progression-free survival (PFS), and overall survival (OS), were evaluated. We also used the Montreal Cognitive Assessment (MoCA) to assess the neurocognitive status changes.

**Results:**

The most frequently observed location of glioma was the frontal lobe (33/80, 41.25%), whereas the tumor primarily invaded the language-related cortex (36/80, 45%). Most patients had supratotal resection (11/80, 13.75%) and total resection (45/80, 56.25%). The median PFS was 43.2 months, and the median OS was 48.9 months in our cohort. The transient (less than seven days) neurological deficit rate was 17.5%, whereas the rate of persistent deficit (lasting for three months) was 15%. At three months of follow-up, most patients (72/80, 90%) had KPS scores > 80. Meanwhile, compared to the preoperative baseline tests, the changes in MoCA scores presented significant improvements at discharge and three months follow-up tests.

**Conclusion:**

Awake brain mapping is a feasible and safe method for treating glioma invading the eloquent cortex, with the benefit of minimizing neurological deficits, increasing EOR, and extending survival time. The results of MoCA test indicated that brain mapping plays a critical role in preserving neurocognitive function during tumor resection.

## Introduction

Balancing maximal tumor removal and neurological functional preservation is always a challenge for patients with glioma infiltrating the eloquent regions of the cortex. Surgical resection is considered the first-line treatment for glioma management, with the benefit of reducing tumor volume and increasing survival time (1). However, when glioma is identified in the eloquent areas, the potential risk of neurological function disturbances increases significantly (2). Therefore, awake surgery, which allows for intraoperative brain mapping of motor-sensory and language functions by directly stimulating cortical and subcortical areas, has been adopted to improve the safety of surgical interventions (3–5). Evidence has suggested that awake surgery could maximize the extent of resection (EOR) of tumors and relieve the symptoms caused by tumor mass effect, particularly in low-grade gliomas (LGG) (2, 6).

More glioma patients benefit from longer survival durations as glioma treatment regimens advance (7). However, glioma patients frequently present with impaired neurocognitive functions, such as memory, language, attention, and executive functions (7–9). Meanwhile, medical providers and patients have paid recent attention to neurological and neurocognitive status (7, 10, 11). Previous research has also indicated that neurocognitive function is an important predictor for glioma patients, providing insight into overall survival (OS), progression-free survival, and further tumor management (12, 13). Therefore, assessing the neurocognitive status is crucial for optimal surgical and oncological management.

The Montreal Cognitive Assessment (MoCA) is a brief screening tool that helps medical service providers make more informed medical decisions to assess a patient’s cognitive health (14, 15). Compared to comprehensive neuropsychological assessment batteries, which are too long and sophisticated for most patients in routine care, MoCA provides a short and sensitive enough tool to detect cognitive impairment, particularly in people with brain metastases (16, 17). However, the utility and feasibility of MoCA for glioma patients receiving awake surgery have seldom been reported (10, 18, 19).

In the present study, we aimed to evaluate the experience of one tertiary neurosurgical center over eight years, performing awake brain mapping for glioma patients using direct cortical and subcortical stimulation to preserve neurological functions. We presented the clinical outcomes and evaluated the effect of awake mapping techniques on perioperative cognitive changes using MoCA tests.

## Methods and materials

### Patients and study design

As an observational retrospective study, we reviewed a cohort of 80 patients who underwent awake surgery with intraoperative direct electrical mapping for dominant and nondominant hemispheres. All patients were treated at Department of Neurosurgery, Tangdu Hospital, Airforce Medical University, from January 2013 to December 2021. The inclusion criteria were (1) age ≥ 18 years, (2) newly diagnosed glioma, including astrocytoma, oligodendroglioma, anaplastic oligodendroglioma, anaplastic astrocytoma, anaplastic oligoastrocytoma, and glioblastoma, based on the WHO 2007 classification. The WHO 2016 classification was applied in 2017-2019 (31 cases), and the WHO 2021 classification of glioma was applied in 2021 (18 cases). The exclusion criteria included biopsy and incomplete MRI data calculating the tumor volume.

Demographic, clinical, and histological data were collected and analyzed from patients and neurocognitive and functional outcomes. The Institutional Review Board at Tangdu Hospital approved the study (TDLL-202210-18).

### Perioperative neuroradiological evaluation

Preoperative MRI imaging (T1 and T2-weighted imaging, diffusion-weighted imaging, with and without gadolinium) was performed one week before surgery. Postoperative MRI (T1 and T2-weighted imaging) was also performed within 72 h to assess the EOR three months later and every three months after that.

The region of interest was delineated manually, and the volumetric analysis was performed according to the thickness of the scanning layer by evaluating pre- and post-operative tumor volumes. EOR was estimated by measuring volumes of perioperative T1-weighted, T2-weighted and T2-fluid-attenuated inversion recovery (T2-FLAIR) images. EOR was defined as follows: (1) supratotal resection, EOR > 100%; (2) gross total resection (GTR), EOR > 95%; (3) subtotal resection (STR), EOR = 85%-95%; and (4) partial resection (PR), EOR < 85% (1).

### Preoperative and postoperative neurocognitive assessment

Basic clinical features of patients were obtained through neurological and physical examinations and Karnofsky Performance Score (KPS). KPS was the most applied tool to assess daily functional status, especially for cancer patients (20).

To minimize the test-retest effect, the Chinese version of MoCA test, including Beijing version of MoCA (MoCA-BJ) and Changsha version of MoCA (MoCA-CS), was used for neurocognitive evaluation to assess patients’ cognitive health (14, 21, 22). All patients were evaluated with MoCA test at three-time points: 48 h before surgery with MoCA-BJ, discharged from the hospital with MoCA-CS, and clinic follow-up three months after surgery with MoCA-BJ. The MoCA test score ranged from 0 to 30, with a higher score indicating better cognitive function. The MoCA test consisted of seven sections: visuospatial/executive (5 points), naming (3 points), attention, concentration and working memory (6 points), language (3 points), abstraction (2 points), delayed recall (5 points), and orientation (6 points). Subjects with scores > 26 were considered cognitively normal. Scores 18-25 indicated mild cognitive impairment, 10-17 indicated moderate cognitive impairment, and < 10 indicated severe cognitive impairment.

### Awake craniotomy and intraoperative mapping tasks

We adopted the asleep-awake-asleep protocol for awake craniotomy with direct brain stimulation, and tumor removal was performed on all 80 patients. After removing the bone flap, the patient was awakened, and cortical mapping was used to identify language and motor areas. The StealthStation S7 neuronavigation (Medtronic Navigation) was applied in each case to plan the surgical incision and identify tumor margins related to brain sulcal and gyral surface structures. Intraoperative ultrasound was also used to help distinguish the tumor boundaries. Before the brain shifts, numerical and letter tags were placed along the cortical tumor margins.

A biphasic current (pulse frequency 60 Hz; single pulse duration 0.5 msec) was delivered through a bipolar stimulator with a 5 mm interelectrode distance for cortical stimulation. The initial setting was 1 mA, gradually increasing the amplitude in 0.5-1 mA increments until reproducible response (motor or sensory function) was obtained or discharge potentials were detected (baseline 1 mA, maximum 8 mA). Stimulation was applied for 4 s at the indicated areas, with a pause of 2-4 s between stimulations. Cortical and subcortical regions were identified using a similar stimulation protocol.

Sensorimotor mapping was first performed to confirm the positive responses (movement and/or paresthesia). Stimulations were repeated at least three times to confirm the positive sites. A negative sensorimotor area was also indicated when no response occurred in the area of interest.

For language mapping, the patient was asked to perform three verbal tasks: counting (regular rhythm, from 1 to 10, repetitively), picture naming (DO80) and word-reading task to identify the essential cortical sites which might be inhibited by stimulation. During the picture naming task, the patient was asked to read a short phrase in Chinese as “this is a ……” before naming each picture to check whether seizures were generated and induced speech arrest if the patient could not name the picture successfully. During the word-reading task, the patient was asked to read Chinese words presented on the computer screen. The duration of each stimulation was also about 4 s. Between each actual stimulus interval, at least one picture was presented without stimulation, and no site was stimulated twice in succession. The types of language disturbances (speech arrest, dysarthria, phonetic/phonemic/semantic paraphasia, anomia, and alexia) found intraoperatively were classified by neuropsychological experts in our department.

By applying the same stimulation parameters, the glioma was removed with alternating resection and electrostimulation for subcortical functional mapping. The patient continuously performed the above tasks throughout glioma resection.

### Outcome evaluation

Each patient receiving awake surgery was followed up, and the primary outcome was postoperative KPS, defined as general daily performance status three months after surgery. Secondary outcomes included OS, defined as the duration from diagnosis to death or most recent follow-up, and PFS, defined as the time from diagnosis to disease progression or the latest follow-up imaging study if no progression occurred.

### Statistical analysis

One-way ANOVA with Bonferroni’s multiple comparisons tests was applied to detect the changes in MoCA total scores and related subdomains. The Kaplan-Meier curves and log-rank tests were used to estimate survival curves. The significance level was set at 0.05, and all tests were performed using SPSS Statistics, Version 25.0 (IBM Corp, Armonk, NY, USA) and GraphPad Prism (version 8.0). The figures were generated by OriginPro 2021 software (OriginLab Corporation, Northampton, MA, USA).

## Results

### Patient demographic characteristics

The present study included 80 glioma patients (45 males and 35 females) who underwent awake surgery from January 2013 to December 2021. **Table 1** summarizes the clinical and demographic information for each patient. The mean age for the awake surgery was 43.84 years (range: 19–68 years). The frontal lobe (n = 33, 41.25%), temporal lobe (n = 12, 15%) and parietal lobe (n = 12, 15%) were the most common tumor locations. Most patients (44/80, 55%) had seizures when admitted to the hospital, and generalized seizures (26/80, 32.5%) were common among them.

**Table 1:** Patient clinical characteristics and demographic features.

Parameters	Value	Percent
Age		
Mean	43.84	–
Median	43.5	–
Range	19-68	–
Sex		
Male	45	56.25%
Female	35	43.75%
Site of lesion		
Right	14	17.50%
Left	66	82.50%
Main tumor location, n (%)		
Frontal lobe	33	41.25%
Temporal lobe	12	15.00%
Parietal lobe	12	15.00%
Insular	10	12.50%
Frontal insular lobe	7	8.75%
Temporal insular lobe	5	6.25%
Frontotemporal insular lobe	1	1.25%
Seizure history, n (%)		
Yes	44	55.00%
No	36	45.00%
Seizure types, n (%)		
Focal seizures	16	20.00%
Generalized seizures	26	32.50%
Auditory/visual hallucinations	2	2.50%
WHO grade, n (%)		
WHO Grade II	43	53.75%
WHO Grade III	18	22.50%
WHO Grade IV	19	23.75%
WHO classification, n (%)		
Diffuse astrocytoma	28	35.00%
Oligodendroglioma	14	17.50%
Oligoastrocytoma	1	1.25%
Anaplastic astrocytoma	9	11.25%
Anaplastic oligodendroglioma	7	8.75%
Anaplastic oligoastrocytoma	2	2.50%
Glioblastoma	19	23.75%
Comorbidities		
Diabetes mellitus	18	22.50%
COPD	13	16.25%
CHD/Hypertension	21	26.25%
Smoker	36	45.00%
Miscellaneous	11	13.75%

WHO, World Health Organization; COPD, chronic obstructive pulmonary disease; CHD, chronic heart diseases. -, not applicable or none.

In our cohort, the most common type of glioma histology was WHO grade II diffuse astrocytoma (23/80, 35%). Glioblastoma (19/80, 23.75%), oligodendroglioma (14/80, 17.5%), anaplastic astrocytoma (9/80, 11.25%), and anaplastic oligodendroglioma (7/80, 8.75%) were the other major pathological types. Based on the development of WHO CNS classification, **Supplementary Table 1** lists the detailed pathological diagnosis with a different version of WHO CNS classification. We summarized the tumor locations by regions in the eloquent cortex by considering the location differences and the relationship between awake surgery and functional outcome. In our case series, the glioma most invaded language-related cortex (36/80, 45%), followed by the premotor cortex (19/80, 23.75%), the primary motor cortex (14/80, 17.50%) and primary sensory cortex (11/80, 13.75%) (**Table 2**).

**Table 2:** Surgical characteristics of patients receiving awake surgery in eloquent regions.

Parameters	Value	Percent
Tumor locations, n (%)		
Primary motor cortex	14	17.50%
Primary sensory cortex	11	13.75%
Premotor cortex	19	23.75%
Language cortex	36	45.00%
Preoperative tumor volume, ml		
Mean (SD)	55.01 ± 67.13	–
Median (IQR)	34.59 (20.56-58.65)	–
Range	0.99-392.1	–
Postoperative tumor volume, ml		
Mean (SD)	3.593 ± 10.82	–
Median (IQR)	0 (0-2.48)	–
Range	0-69.21	–
Extent of resection by volume		
Mean (SD)	51.41 ± 59.57	–
Median (IQR)	31.62 (19.78-54.83)	–
Range	0.99-322.9	–
Extent of resection		
Supratotal resection	11	13.75%
Gross total resection	45	56.25%
Subtotal resection	13	16.25%
Partial resection	11	13.75%
Mapping and surgical adjuncts		
Intraoperative mapping	80	100.00%
Intraoperative ultrasound	71	88.75%
Preoperative KPS, n (%)		
100	62	77.50%
90	13	16.25%
80	4	5.00%
70	1	1.25%
Discharge KPS, n (%)		
100	60	75.00%
90	10	12.50%
80	4	5.00%
70	4	5.00%
60	2	2.50%
3-month follow-up KPS, n (%)		
100	64	80.00%
90	5	6.25%
80	3	3.75%
70	7	8.75%
60	1	1.25%

SD, Standard deviation; IQR, Interquartile range; KPS, Karnofsky Performance Score. -, not applicable or none.

### The extent of resection and outcomes

**Table 2** summarizes the EOR calculated by volumetry. The mean preoperative tumor volume was 55.01 ± 67.13 mm^3^ (range: 0.99–392.1 mm^3^). Compared to the preoperative calculation, postoperative imaging demonstrated a mean residual tumor volume of 3.593 ± 10.82 mm^3^ (range: 0–69.21 mm^3^). The mean EOR measured by volume was 51.41 ± 59.57 mm^3^ (range: 0.99–322.9 mm^3^). According to EOR definition, 11/80 (13.75%) patients achieved supratotal resection, 45/80 (56.25%) patients achieved GTR and 13/80 (16.25%) with STR.

In our cohort, KPS ≥ 80 was considered good functional status, while KPS < 80 was considered a poor outcome. We presented the dynamic changes in KPS at three different time points: preoperative, discharging, and three months follow-ups (**Supplementary Figure 1**). Most patients (79/80, 98.75%) performed well on preoperative KPS. Before being discharged from the hospital, 74/80 (92.50%) patients had an excellent KPS status, and three-month follow-up KPS indicated a similar trend, with 72/80 (90%) patients having KPS scores above 80.

**Figure 1:**
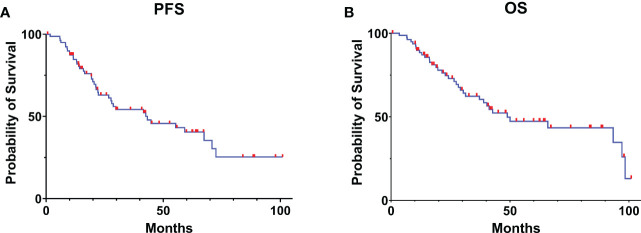
Kaplan-Meier curve estimates of progress-free survival **(A)** and overall survival **(B)** for the patients with glioma receiving awake brain mapping surgery in our cohort.

In our case series, the mean PFS was 43.2 months (95% CI: 16.81-69.58), and the mean OS was 48.9 months (95% CI: 23.17-74.63) in all patients (**Figure 1**).

**Table 3** summarizes the frequency of transient (less than seven days) or persistent (lasting three months) postoperative neurological deficits, such as motor or language disturbance. Due to brain plasticity after traumatic event of the surgery, we still considered the timepoint of three months after surgery follow-up was in the recovery process. During the postoperative period, 11 patients (13.75%) developed new transient speech and language-related deficits, while 3 (3.75%) presented with transient motor-related symptoms. After removing the tumor, 12 patients developed persistent deficits lasting for three months, including five (6.25%) with motor-related disturbance and seven (8.75%) with speech and language disturbance. No cases of persistent speech and language disturbance were reported among patients with tumors located in the parietal and temporal-insular lobes.

**Table 3:** Distribution of patients with transient and persistent neurological deficits.

Impairment	Transient deficit (%)		Persistent deficit (%)	
	No.	Percent	No.	Percent
Motor-related	5	6.25%	3	3.75%
Frontal lobe	2	2.50%	1	1.25%
Parietal lobe	–	–	2	2.50%
Insular	1	1.25%	2	2.50%
Language-related	11	13.75%	7	8.75%
Frontal lobe	3	3.75%	5	6.25%
Parietal lobe	4	5.00%	–	–
Insular	1	1.25%	2	2.50%
Tempo-insular	3	3.75%	–	–
Total	14	17.50%	12	15.00%

Transient deficits: less than seven days; persistent deficits: lasting three months. -, not applicable or none.

### Neurocognitive status with MoCA test

We totally reviewed MoCA test scores from 79 cases, and one case lost post-op and follow-up test. **Table 4** summarizes the changes and distribution of MoCA scores at the preoperative baseline test, discharge from the hospital, and three months follow-up. The total MoCA score increased significantly from baseline to 3-month follow-up (19.95 ± 1.99 vs. 26.65 ± 1.41, p < 0.001). At the three months follow-up visit, most patients (65/80, 81.25%) achieved normal neurocognitive status with MoCA score > 26. At a 3-month follow-up, we found no cases of moderate-to-severe cognitive impairment (MoCA < 20).

**Table 4:** Treatments and neurological deficit and general performance scores.

Parameter	Value	Percent
Postoperative adjuvant therapy		
Radiotherapy only	4	5.00%
Chemotherapy only	16	20.00%
Both radiotherapy & chemotherapy	37	46.25%
With TTF	3	3.75%
None	23	28.75%
Pre-operative MoCA score		
Mean (SD)	19.95 ± 1.99	–
Median (IQR)	20	–
Range	16-26	–
Discharging MoCA score		
Mean (SD)	21.87 ± 1.89	–
Median (IQR)	22	–
Range	18-26	–
3 months follow-up MoCA score		
Mean (SD)	26.65 ± 1.41	–
Median (IQR)	27	–
Range	23-30	–
3 months follow-up MoCA score		
Normal	65	81.25%
Abnormal	14	17.50%
Median progression-free survival, months (95% CI)	43.2	16.81-69.58
Median overall survival, months (95% CI)	48.9	23.17-74.63

TTF, Tumor treating field; MoCA, The Montreal Cognitive Assessment; SD, Standard deviation; IQR, Interquartile range; KPS, Karnofsky Performance Score; CI, Confidence interval. -, not applicable or none.

**Figure 2:**
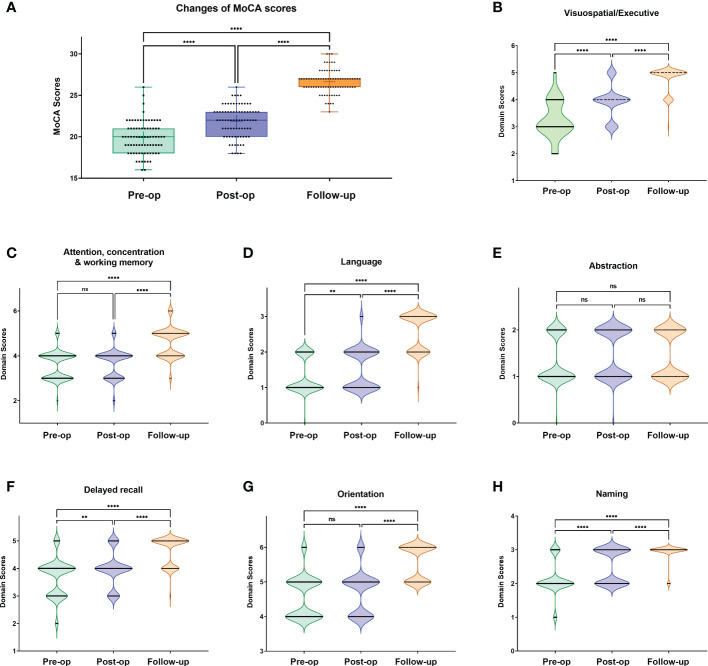
Changes of total MoCA scores **(A)** and subdomain score distribution **(B-H)** at each MoCA test timepoint. One-way ANOVA with Bonferroni’s multiple comparisons tests. **: <0.001; ****:<0.0001; ns, not significant.

We further analyzed the subdomain score distribution for each MoCA test **(** **Figure 2**; **Supplementary Table 2**). With surgical intervention, we noticed that the scores of subdomains were elevated significantly for most domains in each timepoint (post-op *vs.* pre-op, 3-month follow-up *vs.* pre-op, 3-month follow-up *vs.* post-op), including visuospatial/executive, language, delayed recall, and naming domains. For example, in the language domain, the patients demonstrated favorable recovery outcomes as evidenced by post-operative and follow-up assessments. This may be attributed to the measures employed during intraoperative surgical manipulations. However, no significant improvement was observed in the abstraction domain test.

Due to the glioma invading the eloquent area, we divided patients into the functionally related cortex, including the primary motor cortex, primary sensory cortex, premotor cortex, and language cortex (**Supplementary Figure 2**). In general, our findings demonstrated that MoCA scores of patients at three months follow-ups were significantly higher than the baseline MoCA scores in the present cohort. Specifically, for gliomas invading the primary motor cortex and primary sensory cortex, each domain in MoCA test indicated a significant increase from preoperative to three months test (p < 0.001). Whereas, for gliomas invading the premotor and language cortex, most domains in MoCA test presented similar increments at all three-time points, except for the abstraction domain. Although the scores in the domain of abstraction improved in both groups, no significant difference was observed between the preoperative and three months follow-up tests (for premotor cortex, improved score: 0.16 ± 0.69, range:-1~1, p = 0.3306; for language cortex, improved score: 0.00 ± 0.69, range: -1~1, p > 0.99). **Supplementary Table 3** indicates the detailed MoCA scores and subdomain distribution stratified by glioma locations.

In this study, we applied 26 points as the cut-off value to distinguish normal and cognitive impairment cases. We did not observe severe impairment patients but with eight moderate cognitive impairment cases in pre-op tests. To further clarify the relationship between pre-op MoCA status with patients’ clinical features, we established Kaplan-Meier curve and performed the survival analysis. In **Supplementary Figure 3**, we presented PFS and OS for normal and mild cognitive impairments. In **Supplementary Figure 4**, we stratified the WHO pathological categorical classification and presented the OS of each grade glioma case. However, no significant difference was observed in PFS/OS with different MoCA statuses. We also presented the dynamic changes in MoCA scores according to WHO grades (**Supplementary Figure 5**). Based on our current data, although WHO classification grades, including Grade II, III and IV, did not show the survival benefit by MoCA scores stratification, the rate of normal MoCA score was significantly improved, especially at 3-month follow-up test.

## Discussion

Awake brain mapping, a technique for functional preservation, has been widely adopted by neurosurgical institutions in recent years (8, 10, 23, 24). Despite advancements in intraoperative MRI, neuro-navigation systems, and intraoperative imaging techniques, intraoperative direct electrical stimulation for patients with gliomas in eloquent areas remains the gold standard for eloquent cortex localization (25). Studies have indicated that awake brain mapping could improve tumor EOR and OS and reduce the rate of persistent postoperative neurofunctional deficits (6, 26–28). We retrospectively reviewed our institutional experiences with awake surgery in this study. We applied MoCA test to assess the neurocognitive status of patients with glioma in eloquent areas. Our findings confirmed the safety and feasibility of awake surgery in treating gliomas in the eloquent cortex. The awake functional mapping enabled favorable functional and neurocognitive outcomes with MoCA test.

Evidence suggests that EOR > 78% of the contrast-enhanced portion of glioma is an important prognostic factor (29). However, whether awake surgery can improve EOR and OS remains debatable. Gerritsen et al. demonstrated that awake surgery could improve EOR, but the treatment did not affect the patient’s OS (30). Another study found comparable EOR and OS for awake surgery and general anesthesia craniotomy (31). Considering the limited number of cases from previous studies and the difference in technique application between institutions, most surgeons planned to perform the resection based on the preoperative daily status of the patient and intraoperative real-time stimulation feedback. In this study, total resection was achieved in more than half cases (45/80, 56.25%), consistent with previous studies (30, 32).

In addition, supratotal resection (11/80, 13.75%) was achieved for selected cases. Supratotal resection was still defined differently by neurosurgical oncologists. Generally, resection beyond 1-2 cm for contrast-enhanced tumors or 1-2 cm beyond the boundary in Flair images for non-enhanced tumors could be considered acceptable supratotal resection (33). Evidence indicated that supratotal resection might improve EOR and prolong the progression-free as well as OS in glioma patients (34). Our findings also suggested that awake brain mapping enabled surgeons to achieve supratotal resection with favorable neurological and clinical outcomes while preserving the neurocognitive function.

As the primary purpose of brain mapping, neurosurgeons in the operation room always prioritize minimizing the risk of postoperative neurological deficits. Previous studies reported varied morbidity rates. A meta-analysis revealed that with stimulation mapping, the early neurological deficit rate could be 47.8%, and the late neurological deficit rate could be 6.4% (27). Li et al. demonstrated that early and late deficit rates were 19.6% and 10.7%, respectively (35). In contrast, Trinh et al. reported 38% and 13%, respectively (36). This study revealed that the transient deficit rate was 17.5%, significantly lower than previous studies, while the persistent deficit was 15%, which was consistent with most studies.

Notably, language-related deficits accounted for the most significant proportion of transient and persistent morbidities (13.75% and 8.75%, respectively). Considering the similar proportion of glioma in the premotor cortex (35/80) and language cortex (36/80), the differences in morbidity rate between these two groups suggested that awake brain mapping for the language cortex required more sophisticated intraoperative monitoring and evaluation. Meanwhile, previous research indicated that awake surgery with brain mapping could reduce late severe persistent neurological deficits.

This cohort observed early transient speech and motor disturbances in 11/80 and 5/80 patients. In contrast, the late persistent speech and motor disturbance rates were 7/80 and 3/80, respectively. Most transient deficits were recovered within a few weeks after resolving tissue swelling and reducing stress responses. Consequently, awake mapping enabled tumor resection by more precisely identifying the critical structures to avoid persistent functional deficits, which significantly helped intraoperative tumor resection manipulation control (2).

Neurological performance status is a prerequisite for awake mapping (35). Several studies have found that postoperative KPS scores were significantly improved in awake craniotomy than in general anesthesia (35, 37). The preoperative baseline KPS score in the present cohort was good (KPS ≥ 80, 79/80). Most patients returned to their preoperative KPS score after the awake mapping and tumor resection. However, a small portion of cases experienced transient neurological deficits. At the three-month follow-up visit, most cases indicated an improvement in KPS between pre-and postoperative periods, corresponding to the improvement in neurological deficits.

Although MoCA was initially designed for patients with mild cognitive impairment (MCI) and Alzheimer’s disease, the evidence suggests that it is superior to Mini-Mental State Examination (MMSE) in detecting cognitive impairment in patients with brain metastases (38, 39). The sensitivity of MoCA was lower compared to a comprehensive neuropsychological battery in sensitivity to detecting cognitive deficits (40). However, due to the intrinsic nature of a comprehensive battery, the comprehensive battery administration process may take several hours, and the presence of fatigue may interfere with the patient’s performance (41). In addition, the results of comprehensive battery may be affected by the professional expertise and level of experience of the evaluators, limiting the scope of its application as routine tests. Therefore, researchers tried to investigate the use of MoCA in the primary brain tumor population, especially in the setting of global COVID-19 pandemic (16, 42).

In our case series, we identified that MoCA has a surprisingly high sensitivity in neurocognitive impairments detection. The overall MoCA score improvements (preoperative baseline vs. three-month follow-up test) indicated that surgical intervention had a clinical benefit in terms of neurocognitive improvement. In this study, several patients presented with lower baseline MoCA scores (median: 20; range: 16-26; Normal: 1 case; Mild: 70 cases). When the patients were discharged from the hospital, they revealed a trend of improvement (median: 22; range: 18-26; Normal: 1 case; Mild: 79 cases), while a three-month follow-up test demonstrated a significant increase in MoCA score (median: 27; range: 23-30; Normal: 65 cases; Mild: 14 cases; **Supplementary Figure 6**).

Detailed analysis of MoCA domains supported the improvement of cognitive conditions in most cases. For abstraction domain, we did not observe any significant improvement during the whole hospitalization and 3-month follow-up. According to Zhang et al., the scores of memory function and abstract thinking were significantly different presented for grade III glioma patients, and their results implied that patients with IDHwt-astrocytoma/anaplastic astrocytoma are more susceptible to suffering from neurocognitive function decline than those with other subgroups of grade II and III gliomas (43). Our current data did not further support the relationship between the cognitive status of MoCA scores and the pathological classification (**Supplementary Figures 3**–**5**). More concern should be paid to this issue in future work.

Other studies suggested that KPS, age, education, and previous treatment were associated with patient MoCA outcomes and that the cutoff score appropriate for neuro-oncology establishment required further validation (16, 40). Therefore, based on our results, MoCA administration for neurocognitive monitoring was feasible and convenient for patients undergoing awake surgeries.

Since we applied MoCA tests three times during the study, it was inevitable that the test-retest effects could influence the data and results. Several factors might impact the test-retest effect, such as the number of test administrations, test speed, test form, and test-retest interval (44). Considering our current study design, an alternative of applying MoCA test with Beijing version and Changsha version could greatly minimize the retest effect. In addition, though MoCA-Beijing was applied twice, the test interval was almost more than three months. We suggest that this setting up of MoCA tests diminishes the size of retest effect, as the patients are less likely to remember the test contents.

Other new tools for language deficit assessments were reported recently. *El Hachioui et al.* reported ScreeLing application for assessing the presence and severity of aphasia and linguistic deficits 12 days after stroke (45). The ScreeLing aimed at the basic linguistic components (semantics, phonology, and syntax) and has been adopted as an important tool for assessing long-term post-stroke aphasia patients (46) (47). Currently, there is no evidence supporting ScreeLing in language function prediction for glioma patients. Language Screening Test (LAST) was another important screening tool (48), and recently its Chinese version, CLAST, has been developed and reported as an efficient and time-saving bedside aphasia screening tool for stroke patients in the acute phase (49). New evidence also implied that CLAST is suitable for Chinese post-stroke patients with high reliability, validity, sensitivity, and specificity (50). Meanwhile, in the background of Covid-19, TeleLanguage test has drawn special attention. A short telephone-based language test battery for pre- and postoperative language assessments was developed and piloted for 14 brain tumor patients. Preliminary results showed that TeleLanguage battery could provide convenient, optimized patient care and enable longitudinal clinical research (42). For MoCA tests, Jammula et al. also reported a pilot study on the feasibility and utility of telehealth and in-person clinical assessments (16). Considering the unique nature of awake surgery, it is necessary to further evaluate the patients’ language status with more specific tools to better help their neurofunctional recovery process in our future work.

### Limitations

The current study has several limitations. First, the pathological classification of glioma has changed several times due to the time span of the current cohort. Almost half of the cases were diagnosed using previous versions of diagnosis criteria, which correlated with the patient’s prognosis. Second, due to institutional constraints, the comprehensive neurocognitive battery was not applied until 2019, which constrained us to compare the findings of other tests. Third, the test-retest effect was inevitable since MoCA test was performed three times for each patient. We managed to diminish the size of retest effect by adopting two versions of MoCA test. Especially for MoCA-BJ test, the test interval was more than three months. In addition, considering the unique nature of awake surgery, it is necessary to further evaluate the patients’ cognitive status, such as language status, with more specific tools to better help their neurofunctional recovery process in our future work.

## Conclusion

In conclusion, the present study investigated the role of awake functional mapping in the surgical treatment of glioma invading the eloquent cortex. The technique reduced the risk of neurological deficit while providing clinical benefits such as better KPS, increased EOR, and longer survival time. Notably, the postoperative and follow-up neurological and cognitive status on MoCA assessment was improved compared to preoperative status. Our findings demonstrated that awake functional mapping could achieve favorable neurological, neurocognitive, and functional outcomes for glioma patients.

## MoCA statement

The MoCA test is a screening instrument used to facilitate the assessment of mild cognitive impairment. The MoCA copyright permission was obtained for the current research.

## Data availability statement

The raw data supporting the conclusions of this article will be made available by the authors, without undue reservation.

## Ethics statement

The studies involving human participants were reviewed and approved by Ethics Committee at Tangdu Hospital. Written informed consent for participation was not required for this study in accordance with the national legislation and the institutional requirements.

## Author contributions

YuanW and SG wrote the main manuscript text. YuanW and LW designed the study. NW, JL, FC, YZ, YX, and CF conducted the study and collected and analyzed data. YueW, YJ, and WZ provided the service and technical support for this study. PJ and GG supervised this study. NW, LW, and YuanW prepared tables. All authors contributed to the article and approved the submitted version.

## Supplementary material

Supplementary Figure 1 The perioperative changes of KPS for patients with gliomas receiving awake brain mapping surgery at preoperative baseline **(A)**, discharging from hospitalization **(B)** and 3 months follow-up **(C)** evaluations. KPS, Karnofsky Performance Score.

Supplementary Figure 2 The domain distribution and changes of MoCA score in perioperative period and 3 months follow-up, stratified by the glioma functional locations: total changes **(A, B)**, primary motor cortex **(C, D)**, primary sensory cortex **(E, F)**; premotor cortex **(G, H)**; language cortex **(I, J)**. MoCA, the Montreal Cognitive Assessment.

Supplementary Figure 3 Kaplan-Meier curve estimates of progress-free survival **(A)** and overall survival **(B)** for the patients with different preoperative MoCA score status.

Supplementary Figure 4 Kaplan-Meier curve of overall survival for the patients with different preoperative MoCA score status, stratified by WHO glioma grades.

Supplementary Figure 5 The dynamic changes of cumulative MoCA status by different WHO glioma grades.

Supplementary Figure 6 The Sankey diagram of the dynamic flow of neurocognitive status (normal, mild and moderate) by MoCA scores. The number indicates the cases in each MoCA test timepoint.
