# Supplementary figures and images for: The clinical and neurocognitive functional changes with awake brain mapping for gliomas invading eloquent areas: Institutional experience and the utility of The Montreal Cognitive Assessment
Source: Front Oncol. 2023 Feb 22;13:1086118. doi: 10.3389/fonc.2023.1086118 (PMC9992726; doi:10.3389/fonc.2023.1086118)

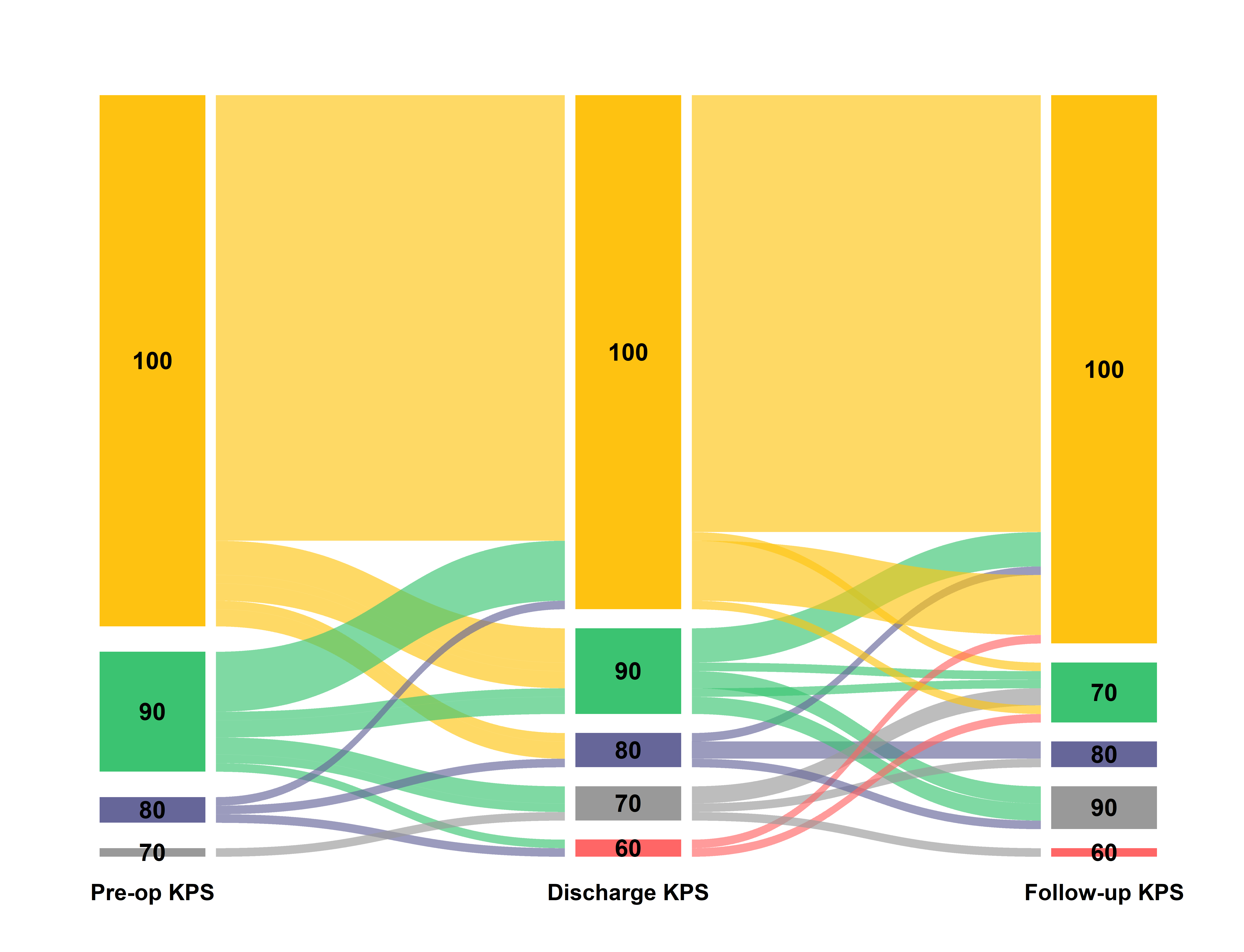

Supplement: Supplementary Figure 1 — The perioperative changes of KPS for patients with gliomas receiving awake brain mapping surgery at preoperative baseline (A), discharging from hospitalization (B) and 3 months follow-up (C) evaluations. KPS, Karnofsky Performance Score. [file Image_1.tif]

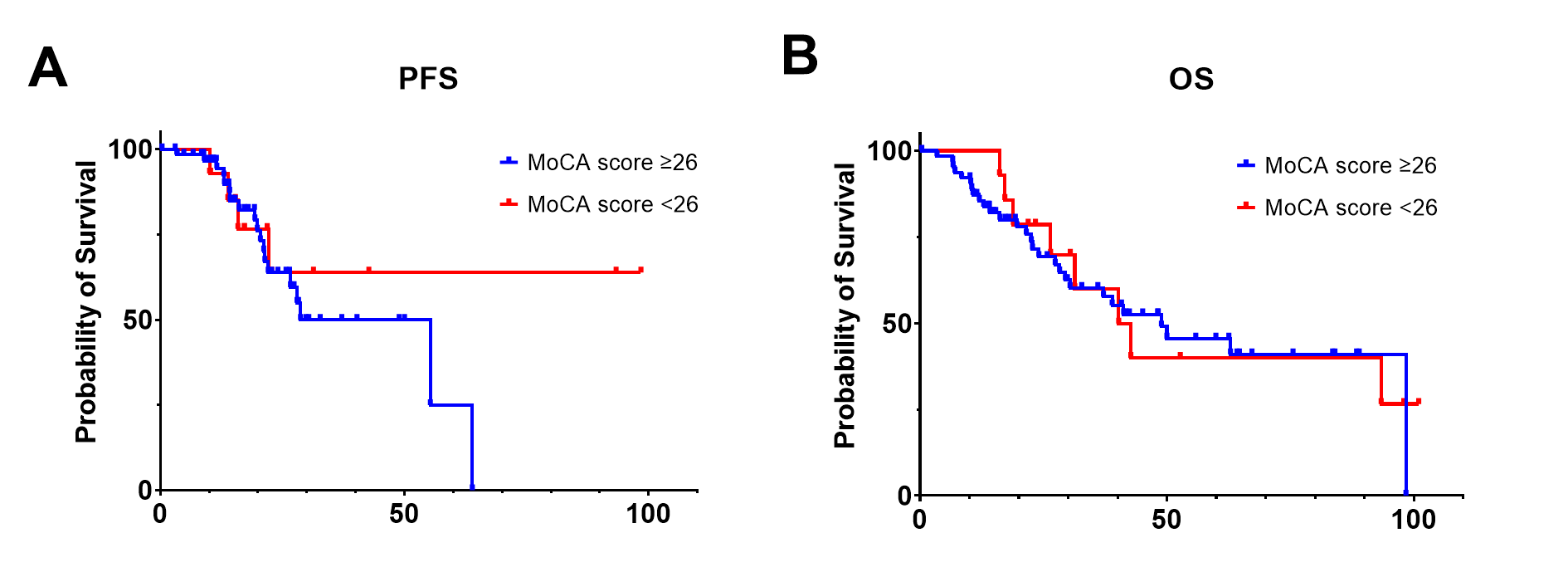

Supplement: Supplementary Figure 2 — The domain distribution and changes of MoCA score in perioperative period and 3 months follow-up, stratified by the glioma functional locations: total changes (A, B), primary motor cortex (C, D), primary sensory cortex (E, F); premotor cortex (G, H); language cortex (I, J). MoCA, the Montreal Cognitive Assessment. [file Image_2.tif]

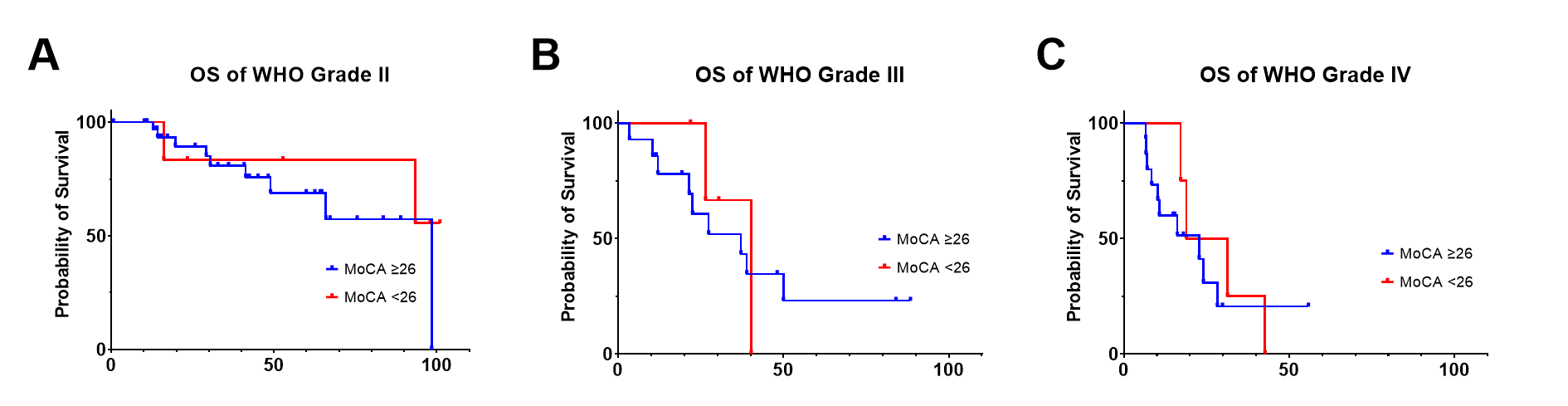

Supplement: Supplementary Figure 3 — Kaplan-Meier curve estimates of progress-free survival (A) and overall survival (B) for the patients with different preoperative MoCA score status. [file Image_3.tif]

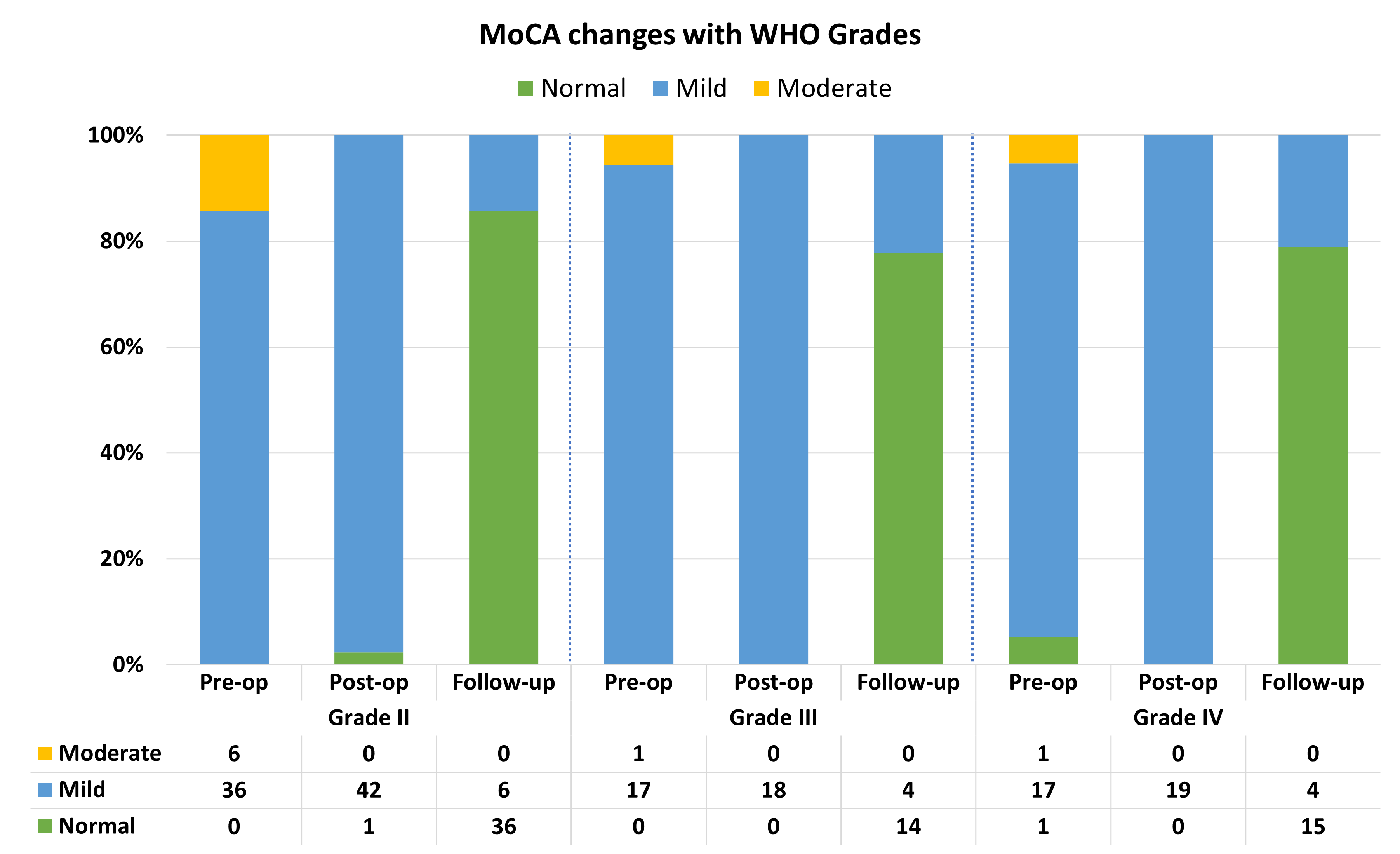

Supplement: Supplementary Figure 4 — Kaplan-Meier curve of overall survival for the patients with different preoperative MoCA score status, stratified by WHO glioma grades. [file Image_4.tif]

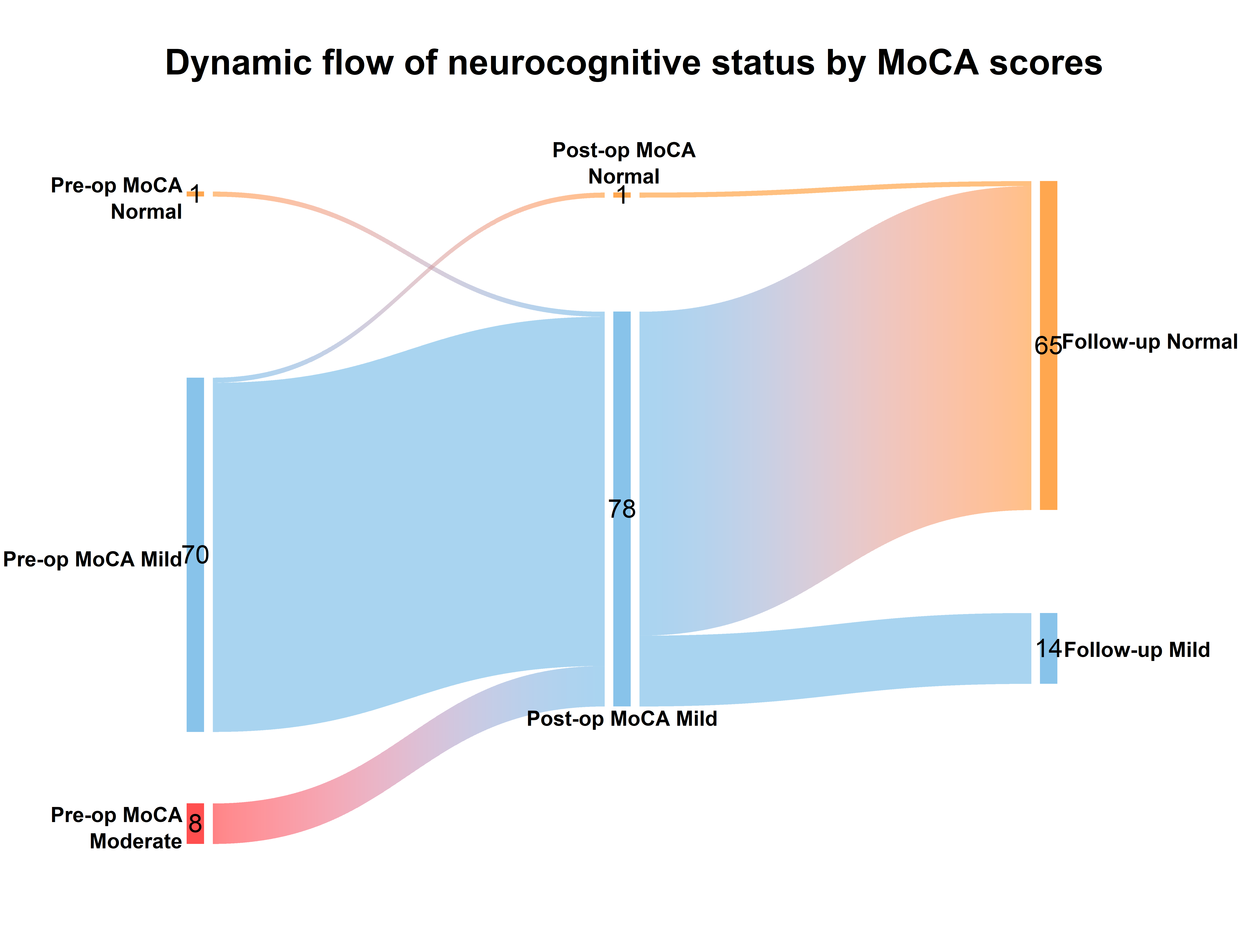

Supplement: Supplementary Figure 5 — The dynamic changes of cumulative MoCA status by different WHO glioma grades. [file Image_5.tif]

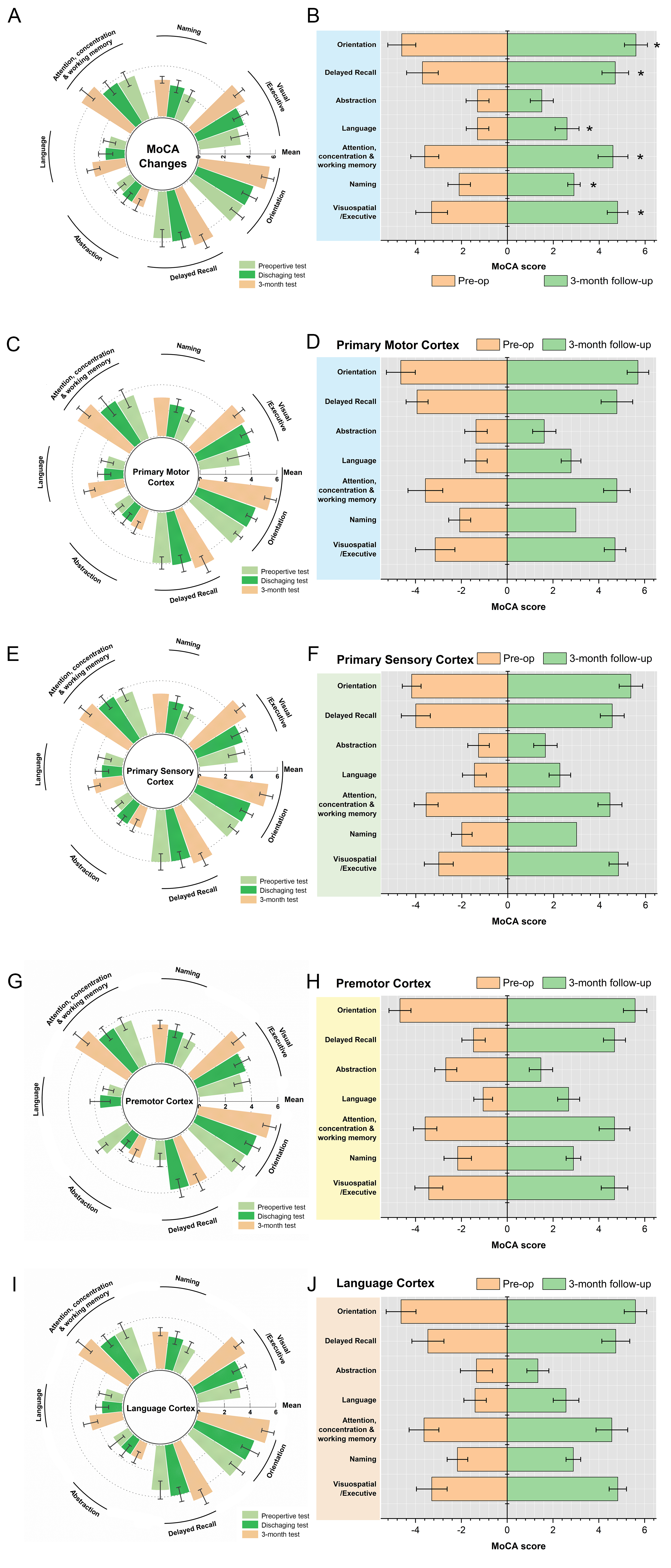

Supplement: Supplementary Figure 6 — The Sankey diagram of the dynamic flow of neurocognitive status (normal, mild and moderate) by MoCA scores. The number indicates the cases in each MoCA test timepoint. [file Image_6.tif]
