# Supplementary material for: The clinical and neurocognitive functional changes with awake brain mapping for gliomas invading eloquent areas: Institutional experience and the utility of The Montreal Cognitive Assessment
Source: Front Oncol. 2023 Feb 22;13:1086118. doi: 10.3389/fonc.2023.1086118 (PMC9992726; doi:10.3389/fonc.2023.1086118)
Supplement: Supplementary file 7 [file Table_1.docx]

**Supplementary Table 1. Pathological diagnosis with versions of WHO CNS classification**

| **Parameter** | **Value** | **Percent** |
| --- | --- | --- |
| **WHO 2007 classification (n=31)** |  |  |
| Diffuse astrocytoma | 10 | 32.26% |
| Oligodendroglioma | 7 | 22.58% |
| Oligoastrocytoma | 1 | 3.23% |
| Anaplastic astrocytoma | 2 | 6.45% |
| Anaplastic oligodendroglioma | 3 | 9.68% |
| Anaplastic Oligoastrocytoma | 2 | 6.45% |
| Glioblastoma | 6 | 19.35% |
| **WHO 2016 classification (n=31)** |  |  |
| Diffuse astrocytoma | 10 | 32.26% |
| Oligodendroglioma | 3 | 9.68% |
| Anaplastic astrocytoma | 7 | 22.58% |
| Anaplastic oligodendroglioma | 1 | 3.23% |
| Glioblastoma | 10 | 32.26% |
| **WHO 2021 classification (n=18)** |  |  |
| Diffuse astrocytoma, WHO Grade 2 | 7 | 38.89% |
| Oligodendroglioma, WHO Grade 2 | 5 | 27.78% |
| Anaplastic oligodendroglioma, WHO Grade 3 | 3 | 16.67% |
| Glioblastoma, WHO Grade 4 | 3 | 16.67% |

**Supplementary Table 2. Subdomain score distribution for each MoCA test**

| **Subdomain scores** | **Pre-op MoCA** | **Post-op MoCA** | **3-month MoCA** | **Pre-op *vs.* Post-op** | **Pre-op *vs.* 3-month follow-up** | **Post-op *vs.* 3-month follow-up** |
| --- | --- | --- | --- | --- | --- | --- |
| Visuospatial/Executive (5 points) | 3.3±0.69 | 3.9±0.61 | 4.8±0.45 | <0.0001 | <0.0001 | <0.0001 |
| Naming (3 points) | 2.1±0.49 | 2.5±0.50 | 2.9±0.27 | <0.0001 | <0.0001 | <0.0001 |
| Attention, concentration and working memory (6 points) | 3.6±0.61 | 3.7±0.63 | 4.6±0.65 | 0.5865 | <0.0001 | <0.0001 |
| Language (3 points) | 1.3±0.49 | 1.5±0.57 | 2.6±0.52 | 0.0014 | <0.0001 | <0.0001 |
| Abstraction (2 points) | 1.3±0.50 | 1.5±0.53 | 1.5±0.50 | 0.2694 | 0.2274 | >0.9999 |
| Delayed Recall (5 points) | 3.7±0.69 | 4.0±0.62 | 4.7±0.58 | 0.002 | <0.0001 | <0.0001 |
| Orientation (6 points) | 4.6±0.61 | 4.7±0.63 | 5.6±0.50 | 0.2301 | <0.0001 | <0.0001 |
| Total score (30 points) | 20±2.0 | 21.87±1.90 | 27±1.4 | <0.0001 | <0.0001 | <0.0001 |

MoCA: The Montreal Cognitive Assessment.

**Supplementary Table 3. Tumor locations and domain distributions of MoCA score**

| **Tumor locations, n (%)** | **Pre-op MoCA** | **Discharging MoCA** | **3-month MoCA** | **Improved score* (mean±SD, range)** | **p** |
| --- | --- | --- | --- | --- | --- |
| **Primary motor cortex, n=14** |  |  |  |  |  |
| Visuospatial/Executive (5 points) | 3.14±0.86 | 4.36±0.49 | 4.71±0.47 | 1.57±0.85 (0-3) | <0.0001 |
| Naming (3 points) | 2.07±0.48 | 2.50±0.52 | 3.00±0.00 | 0.93±0.47 (0-2) | <0.0001 |
| Attention, concentration and working memory (6 points) | 3.57±0.76 | 3.79±0.89 | 4.79±0.58 | 1.21±0.80 (0-3) | <0.0001 |
| Language (3 points) | 1.36±0.49 | 1.5±0.52 | 2.79±0.43 | 1.43±0.65 (0-2) | <0.0001 |
| Abstraction (2 points) | 1.36±0.49 | 1.43±0.51 | 1.62±0.51 | 0.14±0.66 (-1-1) | <0.0001 |
| Delayed Recall (5 points) | 3.93±0.48 | 4.14±0.66 | 4.79±0.69 | 0.86±0.95 (0-3) | 0.1902 |
| Orientation (6 points) | 4.64±0.33 | 4.79±0.43 | 5.71±0.47 | 1.07±0.62 (0-2) | <0.0001 |
| Total score (30 points) | 20.1±2.2 | 22.5±1.79 | 27.3±1.33 | 7.21±1.42 (4-9) | <0.0001 |
| **Primary sensory cortex, n=11** |  |  |  |  |  |
| Visuospatial/Executive (5 points) | 3.00±0.63 | 3.73±0.65 | 4.82±0.41 | 1.82±0.60 (1-3) | <0.0001 |
| Naming (3 points) | 2.00±0.45 | 2.45±0.52 | 3.00±0.00 | 1.00±0.45 (0-2) | <0.0001 |
| Attention, concentration and working memory (6 points) | 3.55±0.52 | 3.82±0.60 | 4.45±0.52 | 0.91±0.94 (0-2) | 0.0096 |
| Language (3 points) | 1.45±0.52 | 1.55±0.52 | 2.27±0.47 | 0.82±0.75 (0-2) | 0.0047 |
| Abstraction (2 points) | 1.27±0.47 | 1.82±0.41 | 1.64±0.51 | 0.36±0.50 (0-1) | 0.0379 |
| Delayed Recall (5 points) | 4.00±0.63 | 4.00±0.63 | 4.55±0.52 | 0.55±0.52 (0-1) | 0.0061 |
| Orientation (6 points) | 4.18±0.41 | 4.36±0.51 | 5.36±0.51 | 1.18±0.60 (0-2) | <0.0001 |
| Total score (30 points) | 19.5±1.75 | 21.7±1.35 | 26.1±1.22 | 6.64±1.80 (4-10) | <0.0001 |
| **Premotor cortex, n=19** |  |  |  |  |  |
| Visuospatial/Executive (5 points) | 3.42±0.61 | 3.95±0.62 | 4.68±0.58 | 1.26±0.81 (0-2) | <0.0001 |
| Naming (3 points) | 2.16±0.60 | 2.53±0.51 | 2.89±0.32 | 0.74±0.65 (0-2) | 0.0001 |
| Attention, concentration and working memory (6 points) | 3.58±0.51 | 3.63±0.59 | 4.68±0.67 | 1.11±0.81 (0-2) | <0.0001 |
| Language (3 points) | 1.05±0.41 | 1.58±0.77 | 2.68±0.48 | 1.63±0.59 (1-3) | <0.0001 |
| Abstraction (2 points) | 1.32±0.48 | 1.58±0.51 | 1.47±0.51 | 0.16±0.69 (-1-1) | **0.3306** |
| Delayed Recall (5 points) | 3.79±0.71 | 3.84±0.69 | 4.68±0.48 | 0.89±0.74 (0-2) | <0.0001 |
| Orientation (6 points) | 4.68±0.58 | 4.84±0.60 | 5.58±0.51 | 0.89±0.32 (0-1) | <0.0001 |
| Total score (30 points) | 20.0±2.05 | 21.9±2.04 | 26.7±1.49 | 6.68±1.86 (3-10) | <0.0001 |
| **Language cortex, n=35 (miss one)** | |  |  |  |  |
| Visuospatial/Executive (5 points) | 3.29±0.67 | 3.83±0.57 | 4.83±0.38 | 1.54±0.85 (0-3) | <0.0001 |
| Naming (3 points) | 2.17±0.45 | 2.54±0.51 | 2.89±0.32 | 0.71±0.57 (-1-2) | <0.0001 |
| Attention, concentration and working memory (6 points) | 3.63±0.65 | 3.63±0.55 | 4.57±0.69 | 0.94±0.91 (-1-3) | <0.0001 |
| Language (3 points) | 1.40±0.49 | 1.54±0.51 | 2.57±0.56 | 1.17±0.75 (0-2) | <0.0001 |
| Abstraction (2 points) | 1.34±0.70 | 1.34±0.54 | 1.34±0.48 | 0.00±0.69 (-1-1) | **>0.99** |
| Delayed Recall (5 points) | 3.46±0.70 | 3.97±0.57 | 4.74±0.61 | 1.29±0.79 (0-4) | <0.0001 |
| Orientation (6 points) | 4.63±0.65 | 4.77±0.73 | 5.60±0.49 | 0.97±0.92 (-1-2) | <0.0001 |
| Total score (30 points) | 19.9±1.92 | 21.6±2.02 | 26.5±1.42 | 6.63±1.96 (3-11) | <0.0001 |

MoCA: MoCA: the Montreal Cognitive Assessment.

SD: Standard deviation.

* Improved score: 3-month follow-up MoCA compared to Pre-op MoCA scores.
